# High risk sexual behaviors are associated with sexual violence among a cohort of women in Durban, South Africa

**DOI:** 10.1186/1756-0500-6-532

**Published:** 2013-12-12

**Authors:** Zakir Gaffoor, Handan Wand, Brodie Daniels, Gita Ramjee

**Affiliations:** 1 HIV Prevention Research Unit, Medical Research Council, Durban, South Africa; 2 The Kirby Institute, Sydney, Australia

**Keywords:** HIV, Sexual violence, South Africa, High risk behaviour

## Abstract

**Background:**

Studies show Gender Based Violence (GBV) to be significantly associated with risky sexual behaviour. In South Africa the incidence of GBV is reportedly high, and there is a strong argument for GBV to be a driver of HIV infection rates. This study describes the prevalence of Forced Sex (FS) experiences of women who enrolled into an HIV biomedical intervention study, and its association with risky sexual behaviour.

**Findings:**

In this study, sociodemographic and behavioural data from women enrolled in the Carraguard™ trial, were assessed in relation to FS using logistic regression. The results indicated that 193/1485 (13%) of women reported ever experiencing FS at the screening visit. Women who were 30 years and older; reported having sex for cash; multiple partners; changing partners during the trial; inconsistent condom use during the trial; and 3 or more sex acts in the 2 weeks prior to screening, were significantly more likely to have experienced forced sex.

**Conclusions:**

The results of this study are broadly consistent with those found in other studies and are similar in profile to women at higher risk for HIV acquisition in our setting. This study indicates a need for GBV prevention to be integrated with HIV prevention programmes.

## Introduction

Thirty three million people are living with HIV worldwide with over half of those infected being women [1]. This figure increases to 60% in sub-Saharan Africa, where young women under the age of 24 years are 2 to 4 times more likely to become infected with HIV than their male peers [1].

Women’s vulnerability to HIV in Sub-Saharan Africa is enhanced through the impact of gender-based violence (GBV) perpetrated by their male partners [2]. The definition of GBV according to the United Nations is ‘Any act of gender-based violence that results in or is likely to result in, physical, sexual or psychological harm or suffering to women, including threats of such acts, coercion or arbitrary deprivations of liberty, whether occurring in public or private life’ [1]. Gender inequality and GBV have been cited by researchers and policy makers as one of the determinants of a woman’s risk for HIV acquisition [3,4]

In South Africa, the high prevalence of GBV and lack of women’s ability to negotiate safe sex is associated with increased risk of HIV acquisition [4-6]. A survey of three provinces showed that 19-28% of women had experienced GBV with 5-7% having been raped. GBV is also associated with high levels of male dominance in a relationship [4]. In the ‘Stepping Stones’ study, rural South African women who experienced GBV had higher gender inequality in their relationships, and an increased HIV incidence [5].

Women who report GBV also report challenges in negotiating safer sex, higher rates of inconsistent condom use, unwanted pregnancies, HIV and other sexually transmitted infections (STIs), multiple sexual partners and early sexual debut [6,7].

A contributing measure of GBV is sexual violence. The latter is defined by specific behaviours as described in the World Health Organizations’ (WHO) multi-country study on women’s health and domestic violence [8]. Given the high prevalence of GBV and women’s inability to negotiate safe sex, there has been a call worldwide to develop women-initiated options for HIV prevention. Vaginal microbicides formulated as gels, films, tablets and rings are designed to be used by women to prevent HIV should she have difficulty in negotiating safe sex.

The Carraguard™ study was undertaken to test a microbicide for prevention of HIV. The study was undertaken in 3 sites in South Africa and screened large numbers of women of reproductive age and at risk for HIV acquisition [9]. Given the interplay between GBV and HIV in this region, it is important to obtain a prevalence estimate of GBV, or components that contribute to GBV, among this key study population. The broader experience of GBV is composed of sexual and physical violence measures, and one aspect of sexual violence is Forced Sex (FS). The latt er is measured in the WHO violence against women instrument, which is a measurement scale of behavior-specific questions designed to collect data about various forms of GBV [8]. One item in the WHO instrument measures FS as being physically forced to have sexual intercourse against their will [8]. Women who report experiencing FS or other sexually violent specific behaviours, may also be at risk for experiencing other forms of violence [2,8]. In view of this, we tested the hypothesis that women who reported ever experiencing FS as a form of GBV in the Carraguard™ study would also be significantly more likely to report risky sexual behaviour.

## Findings

### Methods

#### Study population

The methodology for the Carraguard™ study has been published previously [9]. The study was a phase III multi-site, double-blinded, placebo-controlled trial testing the safety and efficacy of the microbicide Carraguard™, for the prevention of HIV infection in women [9]. Briefly, the main eligibility criteria included being sexually active; HIV negative at screening; willing to provide written consent and follow study procedures; not pregnant with intention to maintain a non-pregnant status; and anticipated residence in the study area for a minimum of 1 year. At all visits, participants received HIV risk reduction counseling and access to condoms.

Women who were HIV-positive at screening were referred to local health care facilities for care and support. Women who seroconverted during the trial remained in the study and were provided with ongoing counseling and referral to local health care facilities for further care upon completion of the studies. All protocols and informed consent forms were approved by the Biomedical Research Ethics Committee (BREC) at the University of KwaZulu-Natal, as well as the various study-specific Institutional Review Boards (IRBs).

#### Data collection

Sociodemographic and behavioural data were collected for all women who presented for screening. The screening data of those who were enrolled into the trial in Durban were analysed (n = 1485), with the outcome variable in our analysis being ‘ever experienced forced sex’. The latter was based on a question in the behaviour questionnaire at the screening visit, which was administered prior to the women receiving risk-reduction counseling [9]. The question was introduced with the following statement: “Sometimes, women are forced to have sex even if they don’t want to”. The question thereafter asked: “Has this ever happened to you with; 1. your steady partner? 2. any of your other partners? 3. anyone else?”. The response options were listed as “yes”, “no” or “refused” for each sub-question. A response of “yes” indicated experience of FS, “no” indicated no experience of FS, and “refused “indicated that the woman did not want to answer the question. Data for FS was collected at this single time-point when the question was asked. Responses to this question did not affect the eligibility of women for enrolment into the study.

#### Statistical analysis

We used STATA Release 10.0 (Stata Statistical Software: Stata Corporation, College Station, Texas, USA) to conduct cross-sectional analysis of the data. Univariate and multivariate logistic regression models were used to determine the independent predictors for FS. Variables including ever abused (physically), partner circumcision status, contraception use at screening, unprotected anal sex in the last 3 months and unprotected oral sex in the last 3 months, were not significant predictors of experiencing FS in the multivariate model (Table 1).

**Table 1:** Univariate and multivariate analysis of demographic and sexual behaviour data, and their association with forced sex from the Carraguard™ trial in Durban

	**N = 1,485 n (%)**	**Univariate analysis OR (95% CI)**	**p-value**	**Multivariate analysis OR (95% CI)**	**p-value**
**Age groups (years)**					
<25	591 (39.80)	1		-	-
25-29	234 (15.76)	1.32 (0.81,2.16)	0.262	1.39 (0.84, 2.30)	0.249
30+	660 (44.44)	2.10 (1.48,2.96)	<0.001	5.60 (2.37, 13.19)	0.000
**Cohabitation status**					
Never married	1,096 (73.80)	1		-	-
Married	389 (26.20)	1.69 (1.23,2.33)	0.001	-	-
**Changed partner during the study**					
No	1,336 (89.97)	1		-	-
Yes	147 (10.03)	1.64 (1.06,2.56)	0.028	1.67 (1.04, 2.66)	0.032
**Coital in last 2 weeks before the screening**					
Less than 3	934 (62.90)	1		-	-
3+	551 (37.10)	1.65 (1.22,2.24)	0.001	1.38 (1.0, 1.90)	0.046
**Ever had sex for cash**					
No	1,444 (97.24)	1		-	-
Yes	41 (2.76)	5.68 (3.00,10.73)	<0.001	3.09 (1.46, 6.53)	0.003
**Ever abused**					
No	1,260 (86.54)	1		-	-
Yes	196 (13.46)	4.53 (3.20,6.42)	<0.001	-	-
**Partner circumcised**					
No	1,135 (76.43)	1		-	-
Yes	350 (23.57)	1.16 (0.82,1.64)	0.412	-	-
**Number of sexual partners, last 3 months**					
1	1,374 (92.53)	1			
2	82 (5.52)	1.67 (0.93,3.00)	0.083	1.43 (0.77; 2.65)	0.249
3 plus	29 (1.95)	9.21 (4.35,19.49)	<0.001	5.60 (2.37, 13.19)	0.000
**Contraception used at screening**					
No	453 (30.51)	1		-	-
Yes	1,032 (69.49)	0.94 (0.68,1.31)	0.722	-	-
**Condom use (last sexual act) at baseline**					
Yes	699 (47.07)	1		-	-
No	786 (52.93)	1.67 (1.22,2.28)	0.001	-	-
**Unprotected anal sex in last 3 months**					
No	1,402 (94.41)	1		-	-
Yes	83 (5.59)	1.65 (0.94,2.92)	0.083	-	-
**Unprotected oral sex in last 3 months**					
No	1,264 (85.12)	1		-	-
Yes	221 (14.88)	1.26 (0.85,1.89)	0.253	-	-
**Condom use reported in%study visits after enrollment**					
100%	334 (22.49)	1		-	-
<100%	1,151 (77.51)	1.92 (1.25,2.94)	0.003	1.98 (1.27, 3.09)	0.003

## Results

Table 1 describes various demographic and behavioural factors that were evaluated for association with FS. Briefly, the study population consisted of 1485 women, of which 13% reported experiencing FS at screening. Approximately 44% were ≥ 30 years of age, and nearly 70% reported contraception use at screening Seventy four percent of women reported never being married. Approximately 92% reported having a single partner in the last 3 months, and 10% reported changing partners during the study. The majority (~76%) reported that their partners were uncircumcised. Thirty seven percent of women reported ≥ 3 sex acts in the 2 weeks prior to screening, and almost 3% reported ever having sex for cash. With regard to condom use, 47% reported at baseline that they had used condoms at the last sex act, whilst approximately 22% reported consistent condom use at all visits post-enrolment. Six percent and 15% of women reported unprotected anal and oral sex in the last 3 months, respectively.

In the univariate analysis, women ≥ 30 years of age were more than twice as likely to report FS (OR 2.10 [1.48, 2.96 95% CI, p < 0.001]. Women who changed partners during the study were significantly more likely to have reported FS at baseline (OR 1.64 [1.06, 2.56 95% CI], p = 0.028). Reporting 3 or more sex acts in the last 2 weeks prior to screening was also significantly associated with more reporting of FS (OR 1.65 [1.22, 2.24 95% CI], p = 0.0001). Women who had sex for cash; and women who reported having 3 or more partners in the past 3 months were strongly associated with reporting FS (OR 5.68 [3.00, 10.73 95% CI, p < 0.001] and OR 9.21 [4.35, 19.49 95% CI, p < 0.001], respectively). Women not using condoms consistently, were almost twice as likely to report experiencing FS (OR 1.92 [1.25, 2.94 95% CI, p = 0.003]).

Experiencing physical abuse; being married; partner circumcision status; contraception use at screening; and unprotected anal and oral sex in the last 3 months, respectively; were also significantly associated with an increased likelihood of experiencing FS in the univariate analysis (ORs at the 95% CI in Table 1) but were not significant predictors of FS in the multivariate model.

In the multivariate model, significant predictors of FS included women ≥ 30 years of age; changing partners during the study; having 3 or more partners in the past 3 months; having 3 or more sex acts in the last 2 weeks prior to screening; sex for cash and inconsistent condom use post-enrolment (OR 5.60 [2.37, 13.19 95% CI, p = 0.000]; OR 1.67[1.04, 2.66 95% CI, p = 0.032]; OR 5.60 [2.37, 13.19 95% CI, p = 0.000]; and OR 1.98 [1.27, 3.09 95% CI, p = 0.003], respectively).

## Discussion

Our findings correlate with previous studies that support women’s experiences of FS as a contributing factor for risky sexual behaviour practices, and therefore HIV acquisition [4,10-13]. A noted difference is the association between older women and an increased likelihood of experiencing FS in our sample, compared to that reported by Abramsky [8], where younger women were more at risk of GBV in twelve of fifteen sites around the world. This could be explained by methodological differences in data collection, since the WHO study, as well as other studies that utilized various measurement scales, accounted for ambiguity in the definition of sexual violence, with FS being just one category that was defined by specific behaviours. [8,13]. Our data may therefore be subject to ambiguity, given that specific behaviours were not defined in the question about FS in our study. It is important to note also, that FS is one aspect in a continuum of GBV measures, therefore FS prevalence cannot be compared with GBV prevalence estimates that incorporate both sexual and physical violence measures. In addition, our analysis arose from a single question asked at a single time point during the screening visit, whereas the WHO study utilised the Violence against Women Instrument for data collection, which measured women’s responses to a much wider range of behaviour-specific questions.

With regard to marital status as a risk factor for the broader experience of GBV, Abramsky [8] noted that formal marriage offered protection across all sites studied. Other reports from South Africa and Uganda respectively, noted no significant associations with marital status and GBV [14,15]. Our report also indicated no significant risk of marital status being associated with FS, whilst bearing in mind methodological differences between studies that may prevent a reliable comparison.

The other factors in our analysis, viz. multiple partners, condom use and sex for cash were also found to be consistent with other studies [3,12,16]. We did not find significant reports in the literature of an association between number of sex acts and reported FS, which we found to be the case in our analysis.

## Limitations

Our analysis had several limitations. Firstly, data was collected from women who presented themselves for screening in an HIV prevention trial. These women were actively recruited based on, among other inclusion criteria, their risk for HIV acquisition. The results may therefore not be generalizable to the local population as a whole. The outcome variable was asked at one time point only in a sexual behaviour questionnaire during the screening process. Therefore, incident analysis of FS during the study time period could not be established. In addition, the questionnaire was interviewer-administered, and responses may have been subjected to social desirability influences. Whilst sensitive behavioural data have commonly been collected using interviewer-administered questionnaires in other studies, there is evidence to support methods such as audio-computer self-interviews and self-administered interviews as tools for yielding data that is less influenced by social desirability [13]. In contrast to other studies in the field, the question of FS in our study was not defined by specific behaviours, which may have contributed to a potentially less rigorous estimate of prevalence and risk factors among our sample population. Lastly, we were unable to obtain data from male partners regarding their experiences with FS, given the nature and design of the clinical trial from which the data was obtained.

## Conclusion

As noted by Wand and Ramjee [17], known risk factors for HIV acquisition in our local population included women in the 25–34 year age group, frequency of sex acts, lack of income, pregnancy incidence, STIs and lack of cohabitation. The study provided a valuable direct measure of FS in a region that is the epicentre of the HIV epidemic in South Africa. The results reported in our analysis show a similar risk profile for women’s experiences of FS, and may provide further evidence of the relationship between HIV risk and FS. This highlights the need for GBV prevention messages to be integrated in HIV prevention programmes in our setting.

## Abbreviations

GBV: Gender based violence; FS: Forced Sex; HIV: Human immune deficiency virus; STIs: Sexually transmitted infections; OR: Odds ratios; CI: Confidence intervals; WHO: World Health Organisation.

## Competing interests

The authors declare that they have no competing interests.

## Authors' contributions

ZG is the lead author and was responsible for project conceptualisation. HW was responsible for experimental design and data analysis, and assisted with project conceptualisation. ZG wrote the manuscript with intellectual input from HW, BD and GR. All authors read and approved the final manuscript.
